# Correlation between ZJU index and sarcopenia in elderly inpatients and its predictive value

**DOI:** 10.3389/fmed.2026.1790722

**Published:** 2026-04-16

**Authors:** Fenfen Liu, Huajie Han, Long Wang, Boyu Tan, Ping Liu, Yujiao Yang, Yi Ding, Ruoling Teng

**Affiliations:** Department of Geriatrics, The Third Affiliated Hospital of Soochow University, Changzhou, China

**Keywords:** inpatients, metabolic indicator, risk assessment, sarcopenia, ZJU index

## Abstract

**Objective:**

To investigate the relationship between the Zhejiang University (ZJU) index and sarcopenia in elderly inpatients, and to evaluate its clinical predictive value.

**Methods:**

This was a retrospective observational study. A total of 205 inpatients aged ≥60 years were enrolled from the Department of Geriatrics, Changzhou First People’s Hospital from January to December 2023. General data, height, weight and laboratory indicators were collected. Appendicular skeletal muscle mass, grip strength and gait speed were measured, and appendicular skeletal muscle mass index (ASMI) was calculated. Patients were divided into non-sarcopenia group and sarcopenia group according to the Asian diagnostic criteria for sarcopenia. Student’s *t*-test, Wilcoxon rank-sum test and χ^2^ test were used to compare clinical data between groups. Spearman correlation analysis and multivariate logistic regression were performed to explore risk factors of sarcopenia, and receiver operating characteristic (ROC) curve was used to assess the predictive performance of the ZJU index.

**Results:**

Among 205 patients, 94 were in the sarcopenia group and 111 in the non-sarcopenia group. Compared with the non-sarcopenia group, the sarcopenia group had significantly lower body mass index (BMI), grip strength, ASMI and 6-meter gait speed, higher prevalence of diabetes mellitus, and statistically significant differences in alanine aminotransferase (ALT), fasting blood glucose (FBG), uric acid (UA) and ZJU index (all *p* < 0.05). Spearman correlation analysis showed a moderate negative correlation between the ZJU index and ASMI. Multivariate logistic regression revealed that a high ZJU index was an independent risk factor for sarcopenia in elderly inpatients (OR = 1.350, 95%CI: 1.218–1.476, *p* < 0.001). ROC curve analysis showed that the area under the curve (AUC) of the ZJU index for predicting sarcopenia was 0.7871 (95%CI: 0.616–0.826).

**Conclusion:**

The ZJU index is an independent risk factor for sarcopenia and has favorable clinical predictive value for sarcopenia in elderly inpatients.

## Introduction

Sarcopenia is an age-related geriatric syndrome characterized by decreased muscle mass, reduced muscle strength and/or impaired physical function (1). Its prevalence increases with age, causing a heavy individual and social burden. Studies have shown that the prevalence of sarcopenia is 5–13% in elderly people aged 60–70 years, and reaches as high as 50% in those aged over 80 years (2). Sarcopenia is often accompanied by physical decline, frailty and varying degrees of disability, which can increase the risk of falls, correlate with elevated mortality, and lead to multiple adverse outcomes (3).

The ZJU index (Zhejiang University index) is a novel comprehensive metabolic scoring parameter established in a Chinese population cohort, initially developed to predict the risk of non-alcoholic fatty liver disease and systemic metabolic disorders. It is constructed by integrating five routine clinical indicators: aspartate aminotransferase (AST), triglycerides (TG), alanine aminotransferase (ALT), body mass index (BMI), and fasting blood glucose. Compared with single biochemical markers, it can more comprehensively reflect the overall status of hepatic metabolism, lipid metabolism, glucose metabolism and body composition, thereby more effectively evaluating the severity of metabolic disorders and the risk trend of related diseases (4). Accumulating evidence has confirmed that the ZJU index is closely associated with non-alcoholic fatty liver disease, obesity, insulin resistance and lipid metabolism disorders (5–7). These metabolic abnormalities are well-established risk factors for the development and progression of sarcopenia, and can accelerate muscle loss and muscle dysfunction through mechanisms including inflammatory pathway activation, aggravated insulin resistance, mitochondrial dysfunction and inhibited protein synthesis. This provides a reliable biological basis for applying the ZJU index to sarcopenia risk assessment. Meanwhile, previous foreign studies have shown that the ZJU index is significantly associated with sarcopenia in adult populations (8), further supporting the extended application of this index in risk evaluation of muscle-related diseases. Compared with single metabolic indicators or metabolic scores developed in foreign populations, the ZJU index has distinct advantages: it was modeled and validated in Chinese populations, making it more consistent with the metabolic characteristics of Chinese people; all included indicators are routine tests in hospitalized patients without additional testing costs, resulting in higher clinical accessibility; as a composite score, it avoids the limitations of large fluctuation and insufficient specificity of single indicators, yielding more stable and reliable evaluation.

Despite these advantages and its reported association with sarcopenia in foreign populations, studies investigating the correlation between the ZJU index and sarcopenia in elderly Chinese inpatients remain extremely scarce. Given the deepening population aging in China, the heavy burden of sarcopenia among elderly inpatients, and the lack of convenient and efficient metabolism-related screening indicators, it is of great clinical significance and practical necessity to conduct research on the ZJU index and sarcopenia in the Chinese population and verify its predictive value in Chinese individuals, so as to improve the early screening system and achieve precise intervention for sarcopenia. Accordingly, this study aims to explore the influencing factors of sarcopenia in elderly inpatients and analyze the correlation between the ZJU index and the risk of sarcopenia, providing evidence for early clinical identification of high-risk populations and the formulation of rational intervention strategies.

## Subjects and methods

### Subjects

**Figure 1:**
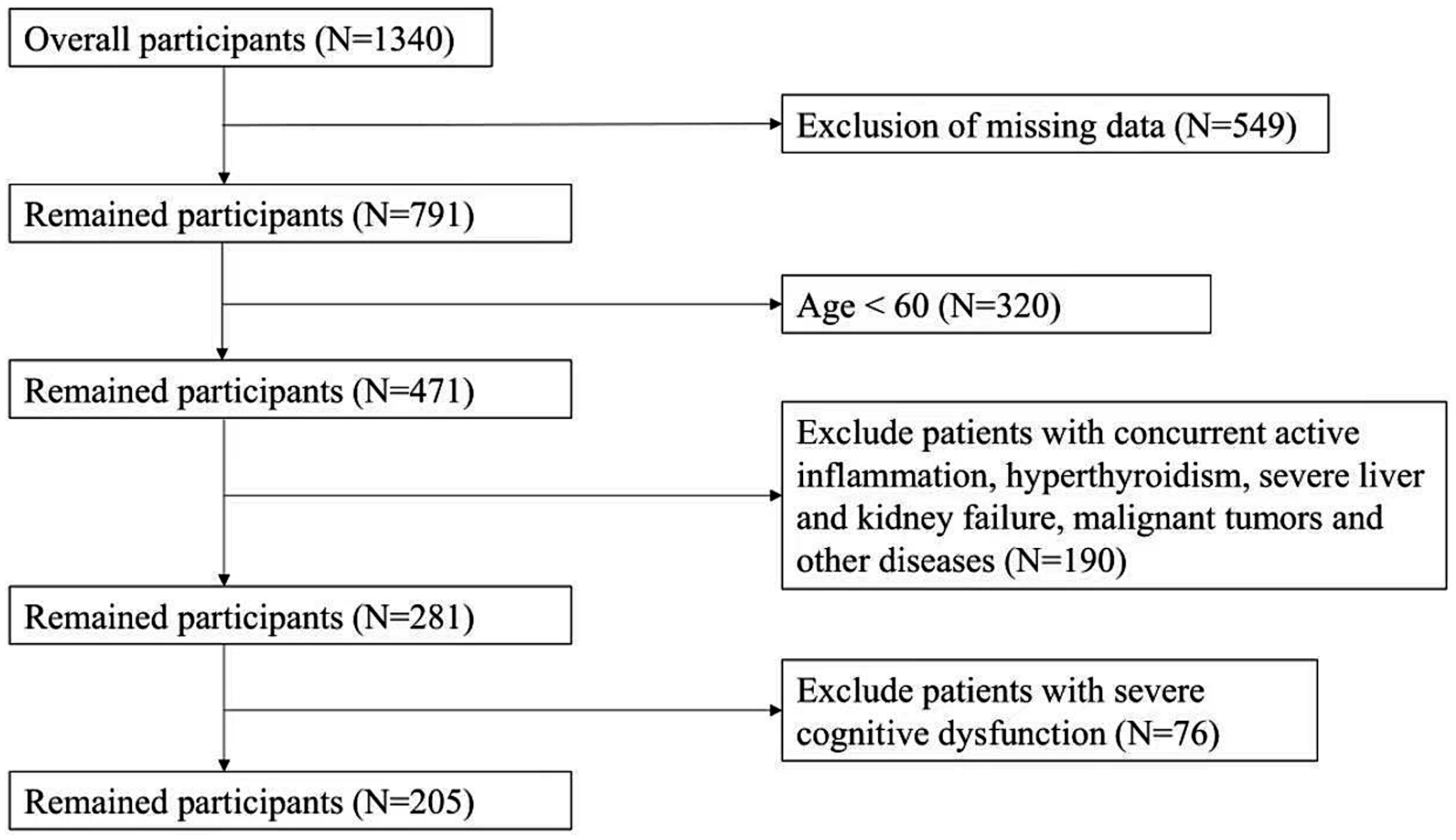
Flowchart of participant selection.

This study enrolled 1,340 inpatients admitted to the Department of Geriatrics, Changzhou First People’s Hospital, from January 2023 to December 2023. The participant selection process is summarized in Figure 1. Exclusion criteria were defined as follows:① Patients who did not undergo sarcopenia-related assessments (e.g., grip strength measurement, gait speed testing) or had incomplete clinical data (including laboratory parameters and causes of hospitalization); ② Patients aged < 60 years; ③ Patients with comorbidities that may significantly affect metabolism and muscle function, such as active inflammation, hyperthyroidism, severe hepatic or renal failure, and malignant tumors; ④ Patients with severe cognitive impairment who were unable to cooperate with the required assessments. Finally, 205 patients with complete data were included in the final analysis. Written informed consent was obtained from all participants prior to their inclusion in the study. This study was approved by the Ethics Committee of Changzhou First People’s Hospital [Approval No.: (2023) Ke Di 073], and all study procedures were conducted in compliance with the Declaration of Helsinki and relevant medical ethical standards.

Given the unique characteristics of hospitalized patients, to control for the potential confounding effects of hospitalization causes and comorbidities on sarcopenia and the ZJU index, the following control and adjustment measures were adopted in this study: ① Comprehensive information regarding the causes of hospitalization (e.g., cardiovascular diseases, respiratory diseases, digestive diseases) and comorbidities (e.g., hypertension, diabetes mellitus, dyslipidemia) was collected for all participants. The causes of hospitalization and underlying comorbidities were regarded as confounding factors and incorporated into subsequent multivariate logistic regression models for adjustment, so as to eliminate their interference with the study results; ② Baseline balance tests (Chi-square test) were performed to compare the causes of hospitalization, as well as the types and severity of comorbidities, between the sarcopenia group and the non-sarcopenia group. This ensured that there were no statistically significant differences in confounding factors between the two groups (*p* > 0.05), thereby reducing the impact of baseline discrepancies; ③ The exclusion criteria were strictly implemented to exclude patients with conditions (e.g., active inflammation, malignant tumors) that may simultaneously affect metabolic indicators (i.e., the components of the ZJU index) and the incidence of sarcopenia, further reducing the confounding effect.

### Research methods

1. *Collection of general data*: Basic data of all research subjects such as age, gender, height and weight were collected, as well as past medical history including hypertension, diabetes, smoking history, drinking history and medication history (e.g., antihypertensive drugs, hypoglycemic drugs, lipid-lowering drugs, etc.). The body mass index (BMI) was calculated.
2. *Detection of laboratory indicators*: Venous blood samples were collected from all patients in the fasting and resting state on the next morning after an 8-h fast and sent for laboratory testing. Indicators including ALT, AST, gamma-glutamyl transpeptidase (GGT), alkaline phosphatase (ALP), lactate dehydrogenase (LDH), albumin (Alb), FBG, blood urea nitrogen (BUN), creatinine (CR), UA, total cholesterol (TC), TG, high-density lipoprotein cholesterol (HDL-C) and low-density lipoprotein cholesterol (LDL-C) were detected. The calculation formula of the ZJU index is as follows: ZJU index = BMI (kg/m^2^) + FBG (mmol/L) + TG (mmol/L) + 3 × (ALT (IU/L)/AST (IU/L)) + 2 (for females) or 0 (for males).
3. *Diagnosis of sarcopenia*: Muscle function assessment included the measurement of grip strength and gait speed. Grip strength was measured using a Jamar hand dynamometer, and patients were asked to perform grip strength tests three times with each hand, with the maximum value used for the study. Gait speed was measured by the 4-meter gait speed test; patients were instructed to walk 4 meters at their usual normal speed from a stationary state at the starting point, the duration was recorded with a stopwatch, and the gait speed (m/s) was calculated. The diagnosis of sarcopenia in this study adopted the 2019 Asian criteria formulated by the Asian Working Group for Sarcopenia (AWGS) (9): (1) Gait speed: the assessment of daily walking speed ≤0.8 m/s; (2) Grip strength: <28 kg in males and <18 kg in females. If either or both were present, bioelectrical impedance analysis was further used to measure muscle mass, including appendicular skeletal muscle mass and total body skeletal muscle mass, and the appendicular skeletal muscle mass index (ASMI) was calculated. ASMI = appendicular skeletal muscle mass (kg)/height^2^ (m^2^). Sarcopenia was diagnosed when ASMI <7.0 kg/m^2^ (in males) or <5.7 kg/m^2^ (in females). The Inbody720 body composition analyzer produced by Biospace Co., Ltd., South Korea was used for the measurement.

### Statistical methods

Statistical analysis was performed using Stata 15.0 software. Continuous variables were expressed as mean ± standard deviation (x ± s), and the Kolmogorov–Smirnov test was used for normality test. Independent samples *t*-test was used for the comparison of normally distributed data between groups, and Wilcoxon rank-sum test was used for non-normally distributed data. Categorical variables were expressed as number and percentage, and the chi-square test was used for intergroup comparison. Spearman correlation analysis and logistic regression analysis were applied to explore the relationship between the ZJU index and sarcopenia and its components, and the optimal cut-off value and AUC (area under the ROC curve) were calculated. A *p*-value <0.05 was considered statistically significant.

## Results

### General data of the research subjects

A total of 205 elderly inpatients were included in this study, with a mean age of (72.95 ± 7.33) years. There were 94 patients in the sarcopenia group and 111 in the non-sarcopenia group. There were no statistically significant differences in age, gender, history of hypertension, AST, GGT, ALP, LDH, Alb, BUN, CR, TC, TG, HDL-C, LDL-C and 5-time chair stand test time between the two groups (*p* > 0.05). Compared with the non-sarcopenia group, the sarcopenia group had a significantly lower BMI (*p* < 0.001), a relatively higher prevalence of diabetes (*p* = 0.015), and statistically significant differences in ALT, FBG, UA and ZJU index between the two groups (*p* < 0.05). The grip strength (*p* < 0.001), ASMI (*p* < 0.001) and 6-meter gait speed (*p* = 0.001) in the sarcopenia group were significantly lower. See Table 1 for details.

**Table 1:** Comparison of clinical data between the sarcopenia group and the non-sarcopenia group [*n* (%) or x ± s].

Index	Non-sarcopenia group (*n* = 111)	Sarcopenia group (*n* = 94)	*p* value
Age (years)	71.54 ± 7.14	74.61 ± 7.25	0.246
Male	45 (40.54)	32 (34.04)	0.338
BMI (kg/m^2^)	23.83 ± 3.32	20.20 ± 4.54	<0.001
History of hypertension	63 (56.76)	44 (46.81)	0.155
History of diabetes	23 (24.47)	45 (40.54)	0.015
ALT (U/L)	14.70 ± 7.40	15.90 ± 10.40	0.023
AST (U/L)	22.10 ± 7.10	21.20 ± 8.30	0.249
GGT (U/L)	21.50 ± 20.90	18.20 ± 12.90	0.053
ALP (U/L)	71.00 ± 29.00	72.00 ± 33.00	0.484
LDH (U/L)	176.00 ± 55.00	181.50 ± 50.00	0.057
Alb (g/L)	41.3 ± 6.40	41.15 ± 5.40	0.885
FBG (mmol/L)	5.26 ± 1.18	5.54 ± 1.97	0.011
BUN (mmol/L)	5.36 ± 1.68	5.21 ± 2.05	0.214
CR (μmol/L)	63.00 ± 18.00	62.45 ± 21.50	0.557
UA (μmol/L)	319.50 ± 96.50	299.30 ± 94.50	0.031
TC (mmol/L)	4.57 ± 1.0	4.56 ± 1.21	0.352
TG (mmol/L)	1.02 ± 0.71	1.03 ± 0.81	0.194
HDL-C (mmol/L)	1.32 ± 0.52	1.42 ± 0.56	0.184
LDL-C (mmol/L)	2.70 ± 0.84	2.62 ± 0.86	0.170
Grip strength (kg)	23.10 ± 11.20	19.25 ± 7.50	<0.001
ASMI (kg/m^2^)	6.45 ± 1.59	4.94 ± 1.20	<0.001
5-time chair stand test time (s)	11.60 ± 5.17	12.90 ± 6.74	0.059
6-meter gait speed (m/s)	1.10 ± 0.90	0.70 ± 0.60	0.001
ZJU index	30.49 ± 6.00	34.72 ± 6.29	<0.001

BMI, body mass index; ALT, alanine aminotransferase; AST, aspartate aminotransferase; GGT, gamma-glutamyl transpeptidase; ALP, alkaline phosphatase; LDH, lactate dehydrogenase; Alb, albumin; FBG, fasting blood glucose; BUN, blood urea nitrogen; CR, creatinine; UA, uric acid; TC, total cholesterol; TG, triglycerides; HDL-C, high-density lipoprotein cholesterol; LDL-C, low-density lipoprotein cholesterol; ASMI, appendicular skeletal muscle mass index.

### Correlation analysis between ZJU index and components of sarcopenia

Spearman correlation analysis was used to analyze the correlation between the ZJU index and components of sarcopenia in elderly inpatients, which indicated a moderate negative correlation between the ZJU index and ASMI (*r* = −0.570, *p* < 0.0001). The results are shown in Table 2.

**Table 2:** Correlation analysis between ZJU index and components of sarcopenia.

Index	Grip strength (kg)	ASMI (kg/m^2^)	5-time chair stand test time (s)	6-meter gait speed (m/s)
ZJU index (*r* value)	−0.060	−0.570	−0.068	−0.065
ZJU index (*p* value)	0.385	<0.001	0.327	0.349

ASMI, appendicular skeletal muscle mass index.

### Univariate and multivariate logistic regression analysis of sarcopenia in elderly inpatients

First, univariate logistic regression analysis was performed, and variables with *p* < 0.05 in the univariate analysis (age, BMI, lymphocyte, FBG, lipoprotein(a) [Lp(a)], diabetes, ZJU index) were included in the multivariate logistic regression model. Then, multicollinearity diagnosis was conducted for the included independent variables, with a variance inflation factor (VIF) > 10 as the criterion for severe multicollinearity. Therefore, BMI and FBG were excluded from the model because they were already included in the calculation formula of the ZJU index. Subsequently, the stepwise backward method (elimination criterion *p* > 0.05) was used to screen variables, and the odds ratio (OR) and its 95% confidence interval (CI) of each factor were calculated. The results showed that the ZJU index (OR = 1.350, 95%CI: 1.218–1.496, *p* < 0.001), Lp(a) (OR = 1.002, 95%CI: 1.000–1.004, *p* = 0.024) and age (OR = 1.056, 95%CI: 1.010–1.104, *p* = 0.017) were independent risk factors for sarcopenia in elderly inpatients.

### ROC curve analysis of ZJU index for predicting sarcopenia in elderly inpatients

**Figure 2:**
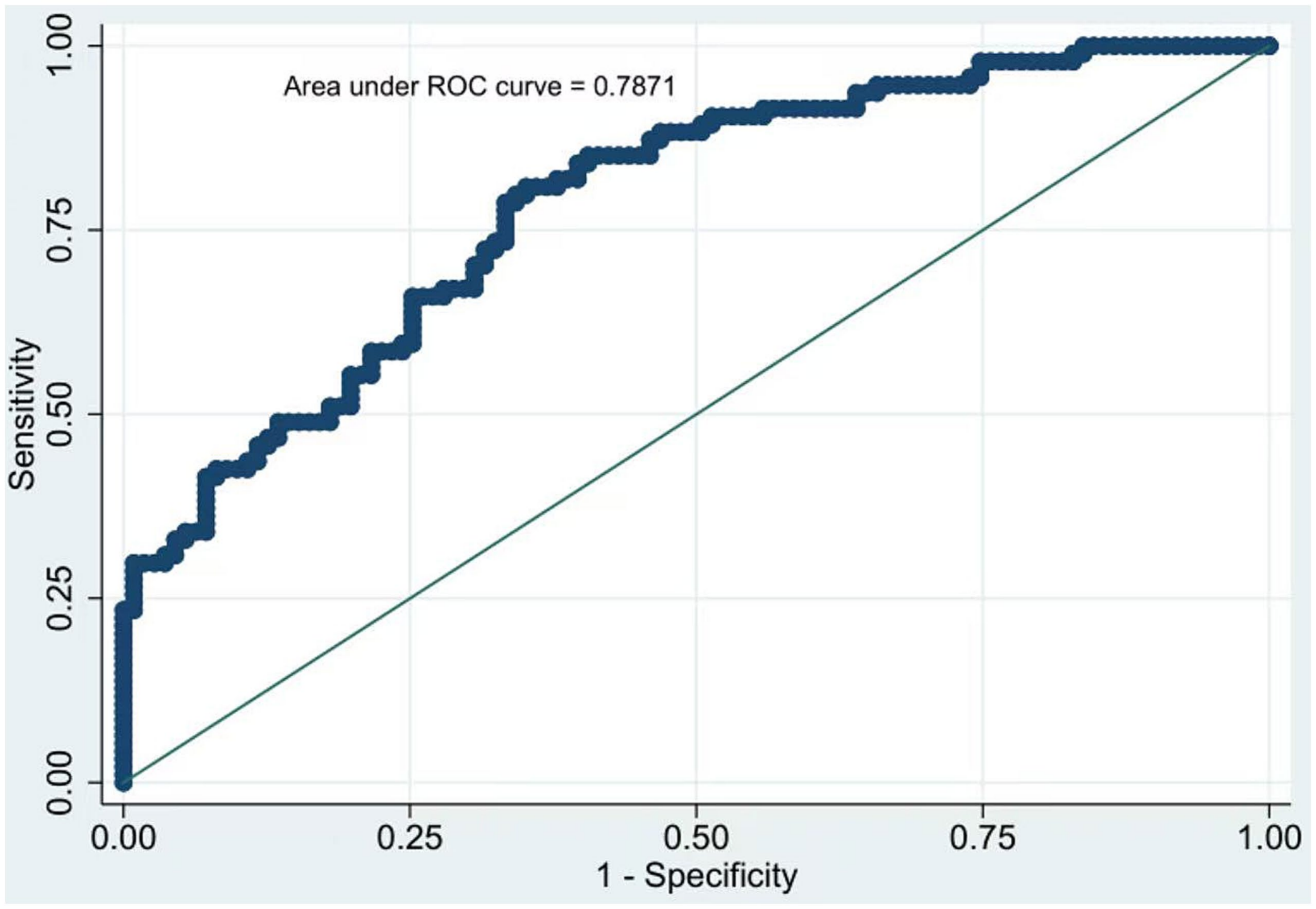
Receiver operating characteristic curve of ZJU index for predicting sarcopenia in elderly inpatients.

The AUC of the ROC curve was calculated, and the AUC of the ZJU index was 0.7871 (95%CI: 0.616–0.826), indicating that the ZJU index had good predictive performance (Figure 2). The diagnostic cut-off value of the ZJU index was calculated to be 31.56. Chi-square test was performed between the ZJU index (<31.56, ≥31.56) and sarcopenia, and the difference was statistically significant (*p* < 0.001).

## Discussion

In the present study, 205 hospitalized elderly patients aged 60 years and older were enrolled to investigate the correlation between the ZJU index and sarcopenia, as well as to evaluate the predictive value of the ZJU index for sarcopenia in this specific population. Our findings demonstrated that the ZJU index was significantly elevated in hospitalized elderly patients with sarcopenia compared with non-sarcopenic controls. Further Spearman correlation analysis revealed a moderate negative association between the ZJU index and appendicular skeletal muscle mass index (ASMI). Multivariate logistic regression analysis confirmed that an elevated ZJU index constitutes an independent risk factor for sarcopenia in hospitalized elderly patients. This finding is consistent with the results derived from the 2011–2018 US National Health and Nutrition Examination Survey (NHANES) (8), while exhibiting distinct population-specific characteristics attributable to inherent differences in the study cohorts. Extraneous studies on community-dwelling middle-aged and young adults (20–59 years) in the United States have also identified a significant positive association between the ZJU index and sarcopenia risk, which supports the potential universal applicability of the ZJU index for sarcopenia risk stratification. Notwithstanding, notable discrepancies exist between these investigations and the present study: the sarcopenia prevalence in the US cohort was merely approximately 5.2%, with a mean ZJU index of 25.38 ± 5.22; in contrast, the sarcopenia prevalence in the present study was 38.24% in patients aged 60–69 years, 47.83% in those aged 70–79 years, and 61.54% in patients aged ≥80 years, with the mean ZJU index increasing in an age-dependent manner to 30.12 ± 5.89, 33.45 ± 6.12, and 36.78 ± 6.35, respectively—all of which were significantly higher than the corresponding values in the US cohort. These disparities are primarily attributable to fundamental differences in age, baseline health status, and ethnic background between the study populations: the study subjects in the present investigation were hospitalized elderly patients with chronic comorbidities, who exhibited both age-related physiological muscle loss and more severe chronic inflammation and metabolic dysfunction secondary to hospitalization. In contrast, the US middle-aged and young cohort displayed stable metabolic profiles, a low prevalence of chronic comorbidities, and far fewer pathophysiological drivers of skeletal muscle loss.

Sarcopenia is highly prevalent in the elderly population, severely compromising physical health and contributing to a spectrum of adverse clinical outcomes. A meta-analysis encompassing 58 independent study cohorts across 26 countries reported a sarcopenia prevalence ranging from 10 to 40% (10), which significantly impairs health-related quality of life and adversely affects disease prognosis. Currently, the diagnosis of sarcopenia is predicated on the assessment of three core dimensions: muscle mass, muscle strength, and physical performance. However, this diagnostic paradigm has not been universally implemented in primary care facilities and geriatric care institutions due to the reliance on specialized detection equipment. Hence, the identification of effective, simple, and rapid biomarkers for the early assessment of sarcopenia represents an unmet clinical need of critical importance. The pathogenesis and progression of sarcopenia are intricately linked to multiple pathophysiological mechanisms, including insulin resistance, chronic low-grade inflammation, and dysregulated lipid metabolism (11). With advancing age, the susceptibility to insulin resistance increases progressively, which suppresses *de novo* muscle protein synthesis and augments proteolysis in senescent muscle tissue, thereby accelerating the rate of muscle mass loss. Age-associated ectopic fat accumulation and increased tissue fibrosis further induce a systemic pro-inflammatory state; chronic low-grade inflammation disrupts cellular architecture and upregulates the expression of pro-inflammatory cytokines, including tumor necrosis factor-*α* (TNF-α), interleukin-1β (IL-1β), and interleukin-6 (IL-6), which in turn impairs skeletal muscle protein anabolism. Additionally, inflammation exacerbates insulin resistance and aberrant fat redistribution, further promoting ectopic fat deposition and subsequent skeletal muscle mass loss (12). Dysregulated lipid metabolism and intramuscular fat infiltration trigger a cascade of pathological events, including sustained inflammation, oxidative stress, insulin resistance, and mitochondrial dysfunction, which collectively contribute to the progressive decline in muscle mass and contractile strength (13).

To date, no direct studies have elucidated the causal relationship between sarcopenia and the ZJU index; however, a growing body of evidence has demonstrated significant associations between sarcopenia and the individual components of the ZJU index. Elevated fasting blood glucose (FBG) is a hallmark of insulin resistance and impaired glucose metabolism, which can trigger systemic inflammatory responses, enhance skeletal muscle protein catabolism, and suppress anabolism. This pathophysiological cascade may further induce microvascular dysfunction in skeletal muscle tissue and compromise the transcellular transport of trace elements (14, 15). A low body mass index (BMI) is indicative of malnutrition and inadequate dietary protein intake, which directly limits the availability of essential raw materials for *de novo* muscle protein synthesis (16). The ratio of alanine aminotransferase (ALT) to aspartate aminotransferase (AST) is a unique component of the ZJU index, and an elevated ALT/AST ratio is strongly associated with non-alcoholic fatty liver disease (NAFLD). Accumulating evidence has confirmed that NAFLD is closely linked to systemic insulin resistance and chronic low-grade inflammation, thereby indirectly impairing skeletal muscle health and function (17, 18). Furthermore, abnormal ALT or AST levels are suggestive of hepatic stress, and impaired hepatic function can disrupt the systemic metabolism and utilization of proteins and anabolic hormones, exerting direct and indirect adverse effects on skeletal muscle tissue (19). Elevated triglyceride (TG) levels are a key indicator of dysregulated lipid metabolism and also a surrogate marker of insulin resistance. Excessive lipid accumulation in skeletal muscle cells (intramuscular fat infiltration) not only impairs muscle contractile function but also induces mitochondrial dysfunction and oxidative stress via the production of toxic lipid metabolic intermediates, ultimately leading to skeletal muscle cell damage and apoptosis (20–22). Collectively, these mechanisms indicate that the ZJU index, as a comprehensive metabolic metric integrating glucose metabolism, nutritional status, hepatic function, and lipid metabolism, modulates the pathogenesis and progression of sarcopenia through the synergistic effects of these multiple interconnected pathophysiological pathways.

We further evaluated the predictive efficacy of the ZJU index for sarcopenia in hospitalized elderly patients using receiver operating characteristic (ROC) curve analysis. The results showed that the area under the ROC curve (AUC) was 0.7871 (95% confidence interval [CI]: 0.616–0.826), with an optimal diagnostic cut-off value of 31.56, indicating favorable predictive performance of the ZJU index for sarcopenia in this population. Two independent studies on American adult populations have also validated the ZJU index as a reliable predictor of sarcopenia in middle-aged adults (8, 23). The present study is the first to confirm the significant correlation between the ZJU index and sarcopenia in hospitalized elderly patients in China, thus providing a convenient and accessible metabolic biomarker for the early screening of sarcopenia in this high-risk population. The findings of the present study hold important clinical implications for the clinical management of sarcopenia in hospitalized elderly patients, with its clinical value manifested in two key aspects. First, it provides a convenient and cost-effective tool for the early screening of sarcopenia: all components of the ZJU index are part of routine laboratory testing for hospitalized patients, incurring no additional diagnostic costs or clinical burden for patients. With its ease of calculation and high clinical accessibility, the ZJU index is particularly suitable for primary care facilities and geriatric care institutions lacking specialized sarcopenia detection equipment (e.g., bioelectrical impedance analyzers, hand grip dynamometers). Clinicians can rapidly calculate the ZJU index to identify individuals at high risk of sarcopenia, thus enabling timely implementation of early intervention strategies. Second, it provides a novel multi-target therapeutic strategy for sarcopenia: the significant association between the ZJU index and sarcopenia confirms that sarcopenia arises from the synergistic dysregulation of multiple metabolic pathways. Therefore, clinical interventions for sarcopenia should not be limited to muscle-targeted nutritional supplementation and exercise training, but should also incorporate strategies to correct systemic metabolic disorders (e.g., glycemic control, lipid-lowering therapy, improvement of hepatic function and nutritional status) to mitigate sarcopenia risk at the source. Additionally, the ZJU index can serve as a dynamic monitoring biomarker, with serial measurements of ZJU index levels used to objectively evaluate the efficacy of therapeutic interventions and provide an evidence-based basis for the individualization and adjustment of clinical treatment protocols.

The present study has several inherent limitations that should be acknowledged when interpreting its findings. First, this is a single-center, retrospective cross-sectional study conducted at the First People’s Hospital of Changzhou, which introduces an inherent regional selection bias; the generalizability of the study results thus needs to be further verified by large-sample, multi-center prospective studies. Furthermore, the cross-sectional study design only confirms a statistical correlation between the ZJU index and sarcopenia, and cannot establish a definitive causal relationship or elucidate the underlying molecular regulatory mechanisms. Second, the study subjects were hospitalized elderly patients with chronic comorbidities, and a subset of patients in the non-sarcopenic group exhibited subclinical impairment of muscle function, rather than being completely healthy community-dwelling elderly individuals. Thus, the predictive efficacy of the ZJU index in healthy community-dwelling elderly populations requires further investigation in dedicated prospective cohort studies. Third, the present study did not perform stratified analyses by age and gender, and the optimal ZJU index cut-off values for sarcopenia prediction in different age and gender subgroups remain to be elucidated. Future studies are therefore needed to refine age- and gender-stratified screening criteria for the ZJU index in the elderly population to improve its clinical applicability.

## Conclusion

The ZJU index is an independent influencing factor for the occurrence of sarcopenia in elderly inpatients, and it can be used as an indicator for early screening and risk assessment of sarcopenia in this population. However, most of the current studies on the ZJU index and sarcopenia are cross-sectional observational studies, which make it difficult to determine the direct causal relationship between them. In the future, more large-scale, multicenter and multi-age randomized controlled trials are needed to verify the causal relationship between the ZJU index and sarcopenia, explore its possible mechanism of action, and formulate potential intervention strategies.

## Data Availability

The original contributions presented in the study are included in the article/supplementary material, further inquiries can be directed to the corresponding author.

## Glossary

BMI: Body mass index
ALT: Alanine aminotransferase
AST: Aspartate aminotransferase
GGT: Gamma-glutamyl transpeptidase
ALP: Alkaline phosphatase
LDH: Lactate dehydrogenase
Alb: Albumin
FBG: Fasting blood glucose
BUN: Blood urea nitrogen
CR: Creatinine
UA: Uric acid
TC: Total cholesterol
TG: Triglycerides
HDL-C: High-density lipoprotein cholesterol
LDL-C: Low-density lipoprotein cholesterol
ASMI: Appendicular skeletal muscle mass index
ROC: Receiver operating characteristic
OR: Odds ratio
CI: Confidence interval
TNF-*α*: Tumor necrosis factor-α
IL-1β: Interleukin-1β
NAFLD: Non-alcoholic fatty liver disease
Lp(a): Lipoprotein(a)
AWGS: Asian Working Group for Sarcopenia
NHANES: National Health and Nutrition Examination Survey
VIF: Variance inflation factor
AUC: Area under the curve
